# The Mouthparts Enriched Odorant Binding Protein 11 of the Alfalfa Plant Bug *Adelphocoris lineolatus* Displays a Preferential Binding Behavior to Host Plant Secondary Metabolites

**DOI:** 10.3389/fphys.2016.00201

**Published:** 2016-06-01

**Authors:** Liang Sun, Yu Wei, Dan-Dan Zhang, Xiao-Yu Ma, Yong Xiao, Ya-Nan Zhang, Xian-Ming Yang, Qiang Xiao, Yu-Yuan Guo, Yong-Jun Zhang

**Affiliations:** ^1^Key Laboratory of Tea Biology and Resources Utilization, Ministry of Agriculture, Tea Research Institute, Chinese Academy of Agricultural SciencesHangzhou, China; ^2^State Key Laboratory for Biology of Plant Diseases and Insect Pests, Institute of Plant Protection, Chinese Academy of Agricultural SciencesBeijing, China; ^3^Department of Biology, Lund UniversityLund, Sweden; ^4^College of Life Sciences, Huaibei Normal UniversityHuaibei, China

**Keywords:** *Adelphocoris lineolatus*, odorant binding protein, expression profiles, phylogenetic analysis, Fluorescence competitive binding assay

## Abstract

Odorant binding proteins (OBPs) are proposed to be directly required for odorant discrimination and represent potential interesting targets for pest control. In the notoriously agricultural pest *Adelphocoris lineolatus*, our previous functional investigation of highly expressed antennal OBPs clearly supported this viewpoint, whereas the findings of the current study by characterizing of AlinOBP11 rather indicated that OBP in hemipterous plant bugs might fulfill a different and tantalizing physiological role. The phylogenetic analysis uncovered that AlinOBP11 together with several homologous bug OBP proteins are potential orthologs, implying they could exhibit a conserved function. Next, the results of expression profiles solidly showed that *AlinOBP11* was predominantly expressed at adult mouthparts, the most important gustatory organ of Hemiptera mirid bug. Finally, a rigorously selective binding profile was observed in the fluorescence competitive binding assay, in which recombinant AlinOBP11 displayed much stronger binding abilities to non-volatile secondary metabolite compounds than the volatile odorants. These results reflect that *AlinOBP11*, even its orthologous proteins across bug species, could be associated with a distinctively conserved physiological role such as a crucial carrier for non-volatiles host secondary metabolites in gustatory system.

## Introduction

Smell is undoubtedly the most important sensory for insects survival and reproduction (Li and Liberles, 2015; Groot et al., 2016). Olfactory system that can sensitively and selectively detect biologically active odorants attracts great attention from researchers who attempt to explore alternative environment-friendly pest management strategy. In insect olfactory signal transduction pathway, several classes of membrane-bound proteins such as odorant receptor (ORs), ionotropic receptors (IRs), and sensory neuron membrane proteins (SNMPs) have been proven to play central roles in facilitating the conversion of the chemical message to an electrical signal, while the carrier proteins like odorant binding proteins (OBPs) or chemosensory proteins (CSPs) are proposed to bind, deliver and even recognize specific pheromones and odorants to their relevant receptors (Jacquin-Joly and Merlin, 2004; Leal, 2013). For decades, various functional studies toward important olfactory protein families such as OBPs or ORs actually lead to a quick discovering of some high-efficiency pest repellents or attractants (Tanaka et al., 2009; Sun Y. F. et al., 2012; Sun L. et al., 2013). For instance, in the alfalfa plant bug, *A. lineolatus*, behavioral active compounds were successfully screened via ligand binding assay of an antennae highly expressed AlinOBP10 (Sun L. et al., 2013). Synthetic compounds targeting OBP3 or OBP7 which was proven to be responsible for (*E*)-ß-farnesene perception elicited significantly behavioral responses in aphids (Sun Y. F. et al., 2012).

Insect OBPs which were first identified in antennal sensillum of silk moth, *Antheraea polyphemus* (Vogt and Riddiford, 1981) belong to the superfamily of small acidic soluble carrier proteins and could be recognized by six highly conserved cysteines (Leal et al., 1999; Sandler et al., 2000; Tegoni et al., 2004; Pelosi et al., 2014). Studies of both immunocytochemical localization and *in situ* hybridization revealed that OBPs were synthesized by non-neuronal auxiliary cells (trichogen and tormogen cells) and secreted into the sensillum lymph with a very high concentration (up to 10 mM) (Steinbrecht et al., 1995; Hekmat-Scafe et al., 1997; Michael, 2000; De Santis et al., 2006; Sun Y. P. et al., 2013; Sun et al., 2014a). So far, various investigations elucidated that antennal sensillum enriched OBPs indeed played essential roles in recognition of physiologically relevant odorants (Jacquin-Joly et al., 2000; Pophof, 2004; Große-Wilde et al., 2006; He et al., 2010). For example, one subclass of OBP families named pheromone binding proteins, PBPs, was highly abundant in long trichoid sensilla and showed significantly specific binding affinities to insect sex pheromones (Vogt and Riddiford, 1981; Krieger et al., 1996; Leal et al., 1999; Klusák et al., 2003; Pophof, 2004; Große-Wilde et al., 2006; De Santis et al., 2006). Sensilla basiconica expressed OBPs were proposed to be involved in terpenoids or other plant volatiles detection (Feng and Prestwich, 1997). Meanwhile, in two Lepidopteran species, the cotton leafworm *Spodoptera littoralis* (Poivet et al., 2012) and the diamondback moth *Plutella xylostella* (Zhu et al., 2016), OBPs were even demonstrated to be associated with the interesting behavior why larvae are attracted by conspecific moth sex pheromone.

However, the functions of insect OBPs may be more complicated and could not be restrict within olfactory cue recognition. In *Drosophila melanogaster*, most OBPs were detected in both gustatory and olfactory sensilla and some numbers were even expressed exclusively in taste organs (Galindo and Smith, 2001). Jeong et al. (2013) proposed that feeding behavior of *D. melanogaster* can be suppressed by a gustatory organ expressed OBP49a responding to bitter compounds. Two OBP genes, *Obp57d* and *Obp57e* in *D. sechellia* have been demonstrated to be involved in the evolution of taste perception and host-plant preference (Matsuo et al., 2007). Particularly, expressions of *Aedes aegypti* OBP22 in antennae and reproductive organs indicated its multiple functions (Li et al., 2008). Likewise, physiological roles of male reproductive organs expressed orthologous OBP10 in two sibling moth species has been proposed to act as a specific carrier for female oviposition deterrents that could help *Helicoverpa* offspring avoid cannibalism (Sun Y. L. et al., 2012).

The alfalfa plant bug, *A. lineolatus*, a typical polyphagous insect pest outbreaks frequently in cotton field since the transgenic *Bacillus thuringiensis* cotton largely cultivation in China (Lu et al., 2010). Worse still, flight behavior enable it to migrate among different host plants (Lu et al., 2009), and many other important crops like alfalfa (*Medicago sativa* L.), green bean (*Phaseolus vulgaris*), and tea plant (*Camellia sinensi*s) suffer from its serious destroy (Lu and Wu, 2008). Evidence suggested that this bug heavily relies on chemical cues for host plant location and migration (Lu and Wu, 2008). Thus, studies aiming at the physiological and molecular basis of insect chemosensation may help explore an alternatively effective pest control method.

Previously, 14 OBP transcripts of *A. lineolatus* were identified (Gu et al., 2011a) and functional studies of several antennae highly expressed OBPs such as AlinOBP1, AlinOBP5, AlinOBP10, and AlinOBP13 indicated their potential olfactory roles (Gu et al., 2011b; Sun L. et al., 2013; Wang et al., 2013; Sun et al., 2014a). Subsequently, Hull et al. (2014) identified 33 putative OBP transcripts in the tarnished plant bug, *Lygus lineolaris*, and suggested that several OBP genes included *LylinOBP19* can be expressed in gustatory organs, implying they may be related to taste compound detected. However, whether OBPs could express at taste organs and fulfill potential gustatory functions in *A. lineolatus* remains largely unknown. In the current study, we mainly focus our attention on *AlinOBP11*, a putative orthologous OBP gene of *LylinOBP19* in *A. lineolatus* and our current results of tissue distribution pattern, ligand binding assay, and phylogenetic analysis would provide detail cues for its functional discussion.

## Materials and methods

### Insect rearing and tissue collection

*A. lineolatus* adults were collected from alfalfa fields at the Langfang Experimental Station of Chinese Academy of Agricultural Sciences, Hebei Province, China. The laboratory colony was established in plastic containers (20 × 13 × 8 cm), which were maintained at 29 ± 1°C, 60 ± 5% relative humidity, and 14 h light:10 h dark cycle. The adults and newly emerged nymphs were reared on green beans and 10% honey. Different tissues from *A. lineolatus* adults of both sexes including antennae, mouthparts, heads (without antennae and mouthparts), thoraxes, abdomens, legs, and wings were collected for qRT-PCR. Each tissue was collected from three biological pools and all the specimens were immediately stored in −80°C for further process.

### RNA isolation and cDNA synthesis

Total RNA of each sample was isolated using the Trizol reagent (Invitrogen, Carlsbad, CA, USA), and the first-strand cDNA was synthesized by FastQuant RT-kit with gDNA Eraser (TianGen, Beijing, China) according to the manufacturer's instructions.

### qRT-PCR

qRT-PCR assay regarding different developmental stages and tissues were carried out using an ABI 7500 Real-Time PCR System (Applied Biosystems, Carlsbad, CA). Two house-keeping genes *Alin*β*-actin* (GenBank No.GQ477013) and *AlinElongation factor* (GenBank No.AEY99651) were used as endogenous controls to normalize the target gene expression and correct for sample-to-sample variation. Taqman primers of *Alin*β*-actin* and *AlinOBP11* cited Gu et al. (2011a) and primers of *AlinElongation factor* were designed using Primer Express 3.0 (Applied Biosystems) and listed in Table S1. For the qRT-PCR reaction, the cDNA was diluted to concentration of 200 ng /μL. Each reaction was performed in a 25 μL mixture of 12.5 μL of Premix Ex Taq (TaKaRa), 1 μL of each primer (10 mM), 0.5 μL probe (10 mM), 0.5 μL of Rox Reference Dye II, 1 μL of sample cDNA (200 ng), and 8.5 μL of sterilized H_2_O. Negative controls were non-template reactions (H_2_O instead of cDNA). The reaction cycling parameters were as follows: 95°C for 10 s, 40 cycles at 95°C for 20 s, 60°C for 34 s. For the data reproducibility, qRT-PCR reaction for each sample was performed in three technical replicates and three biological replicates. Since our preliminary experiment demonstrated that the amplification efficiency between targeted genes and reference gene was similar (data not shown), the comparative 2^−ΔΔCT^ method was used to calculate the relative quantification between tissues (Livak and Schmittgen, 2001).

The comparative analyses of target gene among different tissues and developmental stages were determined using a one-way nested analysis of variance (ANOVA), followed by Tukey's honestly significance difference (HSD) test using the software SPSS Statistics 18.0 (SPSS Inc., Chicago, IL, USA).

### Phylogenetic construction and selective pressure analysis

The 92 OBP sequences of five mirid bug species (GenBank accession numbers and references can be seen in Table S2) were used to infer the evolutionary history with the software MEGA 6.0 with a *p*-distance model and a pairwise deletion of gaps (Tamura et al., 2013). The bootstrap support of tree branches was assessed by re-sampling amino acid positions 1000 times. Estimation of the non synonymous (dN) to synonymous (dS) substitution rate (ω) was performed by the maximum likelihood method (Anisimova et al., 2001) using the Codeml program in the PAML 4.6 package (Yang, 1997).

### Western blot assay

The polyclonal antiserum against the recombinant AlinOBP11 was produced by injecting robust adult rabbits subcutaneously and intramuscularly with the highly purified recombinant protein. Recombinant protein was emulsified with an equal volume of Freund's complete adjuvant (Sigma, St. Louis, MO, USA) for the first time injection (500 μg) and then with incomplete adjuvant for the three additional injections (300 mg each time). The interval between each injection was approximately half a month, and blood was collected 7 days after the last injection and centrifuged at 6000 rpm for 20 min. The serum was purified based on a MAb Trap kit (GE Healthcare) following the manufacturer's instructions. The rabbits were maintained in large cages at room temperature, and all of the operations were performed according to ethical guidelines to minimize the pain and discomfort of the animals.

Crude extracts from different tissues of female and male adult bugs included the antennae, mouthparts, legs, wings, and bodies (without aforesaid parts) were separated on 15% SDS-PAGE, respectively. Samples were transferred to a polyvinylidene fluoride membrane (PVDF, Millipore, Carrigtwohill, Ireland) at the condition of 200 mA for 50 min, and then membrane was blocked using 5% dry skimmed milk (BD Biosciences, San Jose, CA, USA) in phosphate-buffered saline (PBS) containing 0.1% Tween-20 (PBST) for 2 h at room temperature. After washing three times with PBST (10 min each time), the blocked membrane was incubated with purified rabbit anti-AlinOBP11antiserum (dilution 1:2000) for 1 h. Three times washing with PBST again, the membrane was incubated with anti-rabbit IgG horseradish peroxidase (HRP) conjugate and HRP-streptavidin complex (Promega, Madison, WI, USA) at a dilution of 1:10000 for 1 h. The membrane was then incubated with the western blot substrates of the enhanced chemiluminescence western blot kit (CoWinbiotech, China), and the bands were visualized by exposing to X-OMATBT films (Kodak, New York, USA).

### Fluorescence competitive binding assay

The recombinant protein expression and purification was performed according to our previous protocols (Sun L. et al., 2013; Sun et al., 2014a). Briefly, the plasmid containing *AlinOBP11* gene was constructed and transformed into *Escherichia coli* BL21 (DE3) competent cells for recombinant protein expression, and the protein was largely induced with 1 mM isopropyl ß-D-1-thiogalactopyranoside (IPTG) at 37°C for 3–6 h. The purification was performed using two rounds of Ni ion affinity chromatography (GE-Healthcare), and the His-tag was removed with recombinant enterokinase (Novagen). The highly purified proteins were desalted through extensive dialysis, and then the size and purity of the recombinant proteins were verified by 15% SDS-PAGE.

For the ligand binding assays, 45 compounds include 41 volatiles and four non-volatiles were selected based on previously reported isolation from *A. lineolatus* host plants (Meisner et al., 1977; Halloin, 1982; Aldrich, 1988; Loughrin et al., 1995; Röse and Tumlinson, 2004; Millar, 2005). The binding assay was performed on an F-380 fluorescence spectrophotometer (Tianjin, China) at room temperature (25°C) with a 1-cm light path quartz cuvette and 10-nm slits for both excitation and emission. The excitation wavelength was 337 nm, and the emission spectrum was recorded between 390 and 460 nm. Firstly, the constant of AlinOBP11 with the fluorescent probe N-phenyl-1-naphthylamine (1-NPN) was measured, a final concentration of 2 μM protein solution in 50 mM Tris-HCl (pH 7.4) was titrated with aliquots of 1 mM 1-NPN dissolved in methanol to final concentrations ranging from 1 to 16 μM. Then the affinities of other ligands were tested through competitive binding assays using 1-NPN as the fluorescent reporter at a concentration of 2 μM, and the concentration of each competitor ranged from 2 to 30 μM. The fluorescence intensities at the maximum fluorescence emission between 390 and 460 nm were plotted against the free ligand concentration to determine the binding constants. The bound chemical was evaluated based on its fluorescence intensity with the assumption that the protein was 100% active with a stoichiometry of 1:1 (protein: ligand) saturation. The binding curves were linearized using a Scatchard plot, and the dissociation constants of the competitors were calculated from the corresponding IC_50_ values based on the following equation: Ki= [IC_50_] / (1+ [1-NPN]/K_1−NPN_), where [1-NPN] is the free concentration of 1-NPN and K_1−NPN_ is the dissociation constant of the complex protein/1-NPN.

## Results

### Phylogenetic tree construction and selective pressure analysis

**Figure 1:**
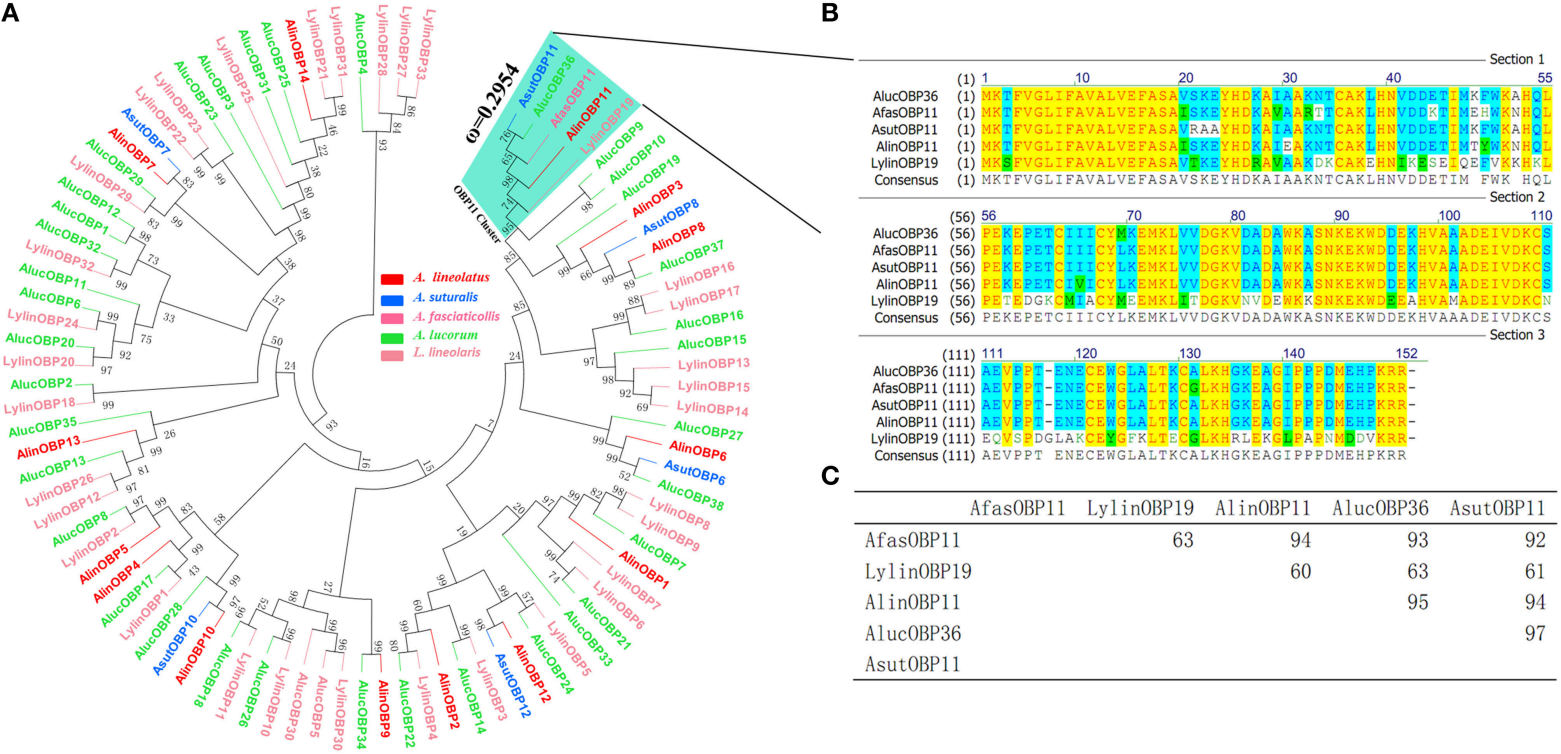
**Sequence alignment and phylogeny of AlinOBP11 with other OBPs identified in five bug species**. **(A)** Neighbor-joining phylogenetic tree was constructed used MEGA 6.0 with a p-distance model and a pairwise deletion of gaps; The non-synonymous (dN) to synonymous (dS) substitution rate (ω) of OBP11 orthologs was labeled beside its cluster. **(B)** Sequence alignment was performed using the program ClustalX 2.1 with default gap penalty parameters of gap opening 10 and extension 0.2, and was edited using the GeneDoc 2.7.0 software. **(C)** The percent identity matrix of OBP11 orthologs is calculated using Vector NTI 10.0.

A phylogenetic tree of 92 OBPs was constructed using the neighbor-joining method to analyze evolutional relationships between AlinOBP11 and other OBPs of different mirid species. Figure 1 revealed a divergent OBP repertoire. AlinOBP11 and four other OBPs i.e., AlucOBP36, AfasOBP11, AsutOBP11, and LylinOBP19 from each bug species clustered into one same clade with bootstrap support value up to 74 (Figure 1A). Sequence alignment analysis showed AlinOBP11 has 94, 95, 94, and 60% identity to AsutOBP11, AlucOBP36, AfasOBP11, and LylinOBP19, respectively (Figures 1B,C). These results reflect AlinOBP11 and these four OBPs form a clear orthologous group across bug species.

To evaluate potential selective pressure acting on this *OBP11* orthologous cluster, we calculated the ratio of non synonymous to synonymous substitutions (dN/dS or ω) of this cluster with branch models using PAML and compared the log likelihoods (lnL) for the one ratio model M0 (assuming one ω ratio for all branches) and the free ratio model M1 (assuming one ω ratio for each branch) in likelihood ratio tests. The results uncovered that the one ratio model (M0) could not be rejected (*p*>0.01) and all branches shared a normalized ω ratio of 0.2954 (Figure 1A), implying that purifying selection was acting on this cluster and *AlinOBP11* would share a relatively conserved physiological function with its orthologous genes (Qiao et al., 2009; Zhou et al., 2010; Vandermoten et al., 2011).

### Specific tissue and developmental expression profiles of *AlinOBP11*

**Figure 2:**
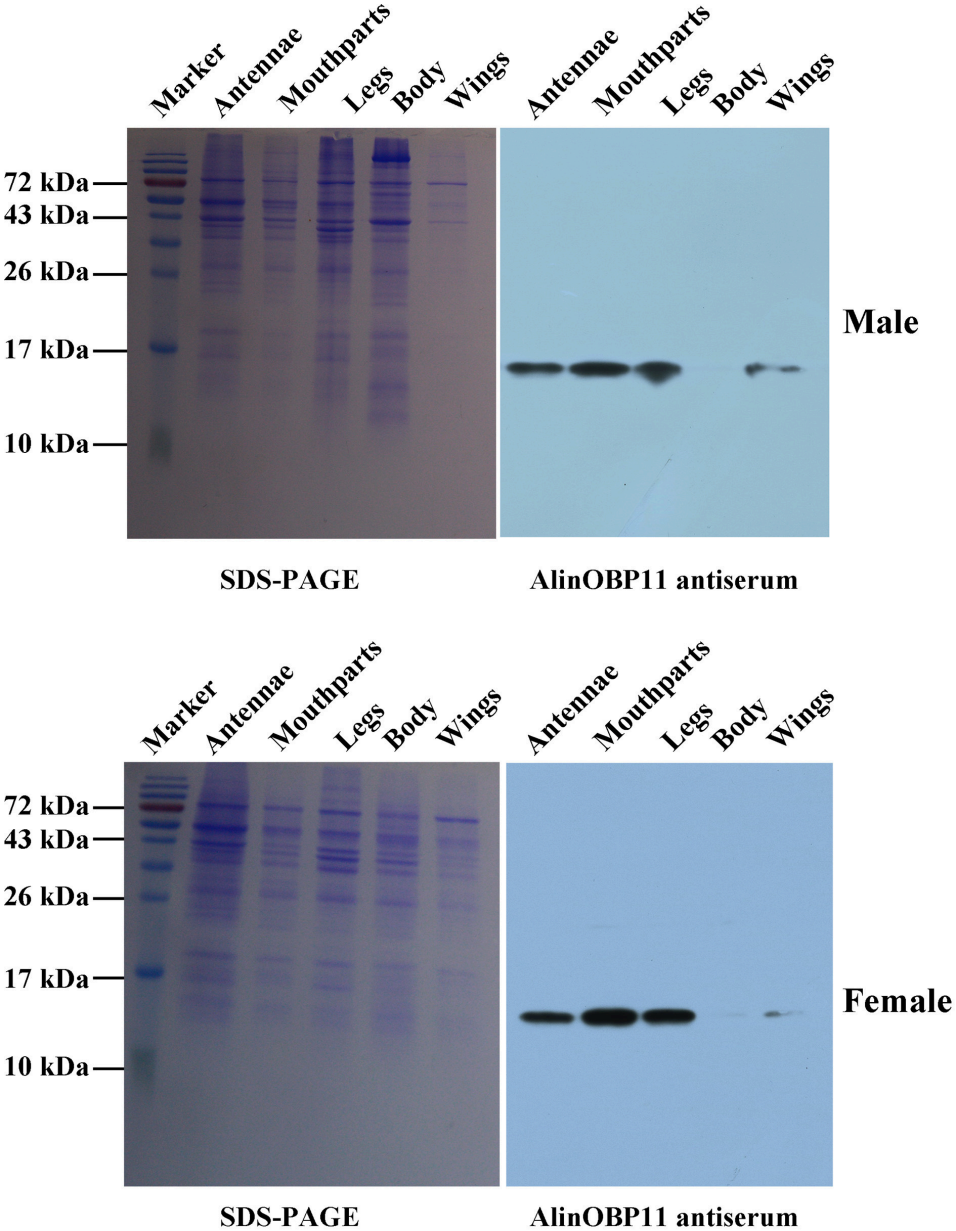
**SDS-PAGE and AlinOBP11 expression profiles among different adult tissues of both sexes assessed by western blot analysis**. The results showed that AlinOBP11 was detected at both male and female adult mouthparts, legs, antennae, and weakly or even undetectably at other tissues.

**Figure 3:**
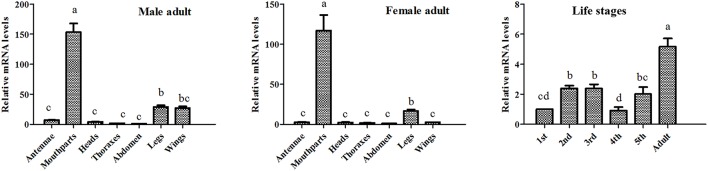
**The relative transcript levels of ***AlinOBP11*** at different developmental stages and adult tissues of both sexes analyzed by qRT-PCR**. All data were normalized to endogenous house-keeping genes *Alin*β*-actin* (GenBank No. GQ477013) and relative fold changes are normalized to transcript level of the first instar nymph or abdomen. The error bars represents the standard errors, and the different letters a, b, c, and d indicate significant differences (*p* < 0.05) among different samples. The similar results were obtained with *AlinElongation factor* (GenBank No. AEY99651) as internal control (Figure S2).

The results of our western blot assay showed that clear protein bands could be found at mouthparts, legs, antennae as well as other tissues, which seems that AlinOBP11 can be ubiquitously expressed at adult tissues of both sexes (Figure 2). To compare the expression levels of *AlinOBP11* among different tissues, we then conducted the qRT-PCR assay. Interestingly, unlike previously reported uniformly antennae predominant expressed *AlinOBPs* (Gu et al., 2011a; Sun L. et al., 2013; Sun et al., 2014a), our current results revealed that *AlinOBP11* was strongly expressed at mouthparts, and slightly expressed at legs, antennae, and other tissues (Figure 3). Meanwhile, *AlinOBP11* transcript abundance varied among different developmental instars and significantly higher expression level was observed in adult bugs (Figure 3).

### *In vitro* expression and purification of AlinOBP11

The recombinant AlinOBP11 was successfully expressed using a bacterial system. Induced targeted recombinant appeared at both supernatant and insoluble inclusion bodies and the former was selected to be purified using two rounds of Ni ion affinity chromatography (GE Healthcare, Little Chalfont, UK). The finally purified AlinOBP11 recombinant protein on the sodium dodecyl sulfate polyacrylamide gel electrophoresis (SDS-PAGE) analysis displayed a single band (Figure S1).

### Ligand-binding of recombinant AlinOBP11

**Figure 4:**
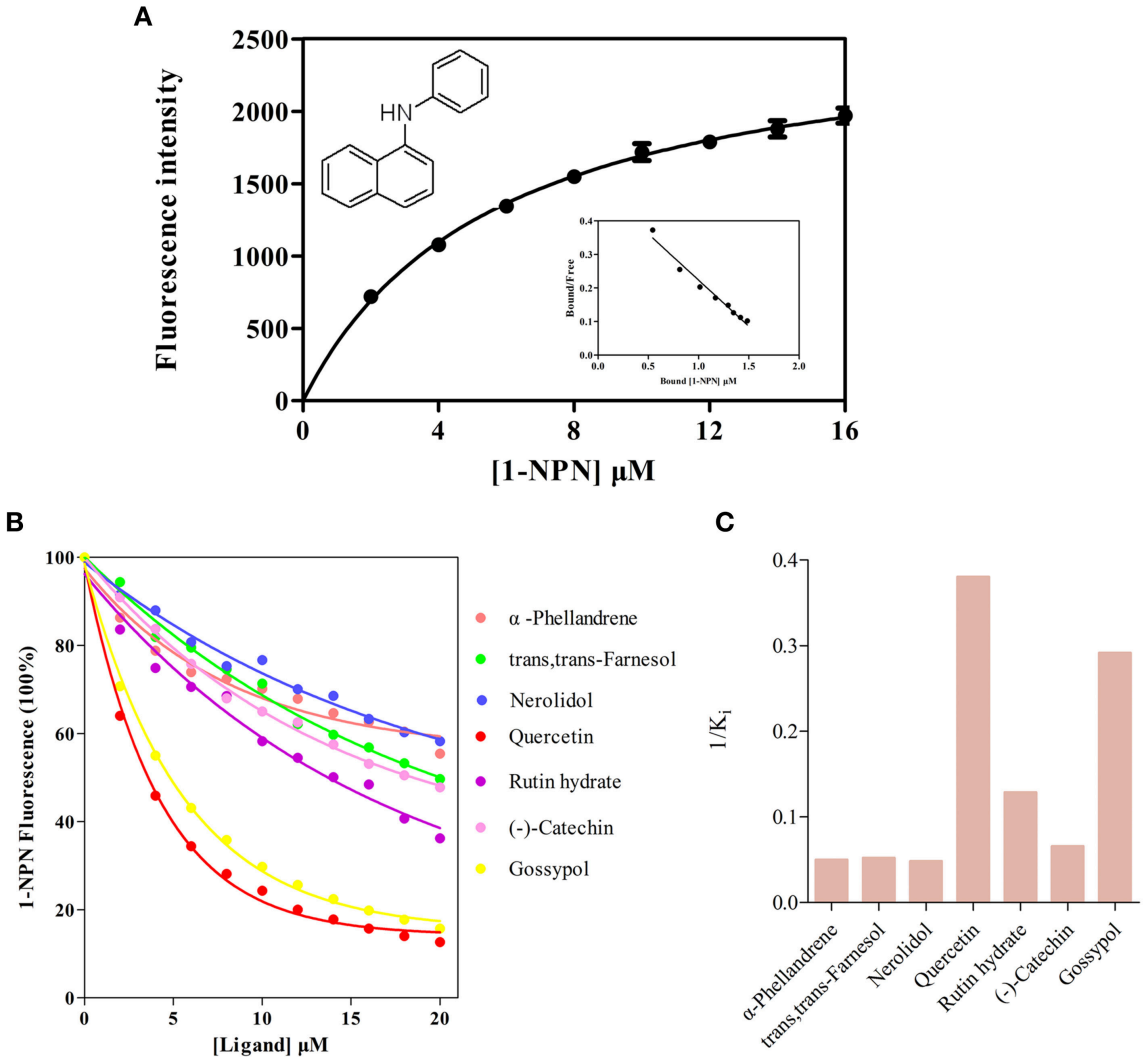
**Fluorescence competitive binding assay**. **(A)** Binding curve and relative Scatchard plot of 1-NPN to AlinOBP11. The dissociation constant of the AlinOBP11/1-NPN complex was calculated as 5.86 ± 0.47 μM. **(B)** Competitive binding curves of selected host plant compounds to AlinOBP11. **(C)** The reverse values of the dissociation constants (Ki) measured with putative ligands of AlinOBP11. A mixture of the recombinant AlinOBP11 protein and N-phenyl-1-naphthylamine (1-NPN) in 50 mM Tris-Hcl buffer (pH 7.4) both at the concentration of 2 μM was titrated with 1 mM solutions of each competing ligand to the final concentration range of 2 to 30 μM. Fluorescence intensities are reported as percent of the values in the absence of competitor. Data are represented as means of three independent experiments.

Before the ligand-binding analysis, we measured the binding affinities of fluorescence probe 1-NPN with purified AlinOBP11. The results showed AlinOBP11 could solidly bind to 1-NPN with binding affinity of 5.86 ± 0.47 μM (Figure 4A). Consequently, the binding properties of AlinOBP11 to compounds with different functional groups were analyzed and the results suggested it had a relatively narrow binding profile. Notably, all the tested non-volatile compounds showed strong binding abilities to AlinOBP11, and quercetin was the best ligand (K_i_ = 2.63 ± 0.23 μM), followed by gossypol (K_i_ = 3.43 ± 0.32 μM), rutin hydrate (K_i_ = 7.78 ± 1.23 μM), and (−)-catechin (K_i_ = 15.26 ± 0.70 μM). Additionally, the tested host volatiles such as aliphatic alcohols, aldehydes, ketones, esters, aromatics could hardly bind to recombinant AlinOBP11, except of three terpenoids α-phellandrene, nerolidol, and *trans, trans*-farnesol, which can bind to AlinOBP11 and their binding constant K_i_ was 20.07 ± 0.41, 20.76 ± 0.55, and 19.26 ± 1.78 μM, respectively (Figures 4B,C; Table 1).

**Table 1:** **Binding affinities of all of the selected compounds to the recombinant AlinOBP11 protein**.

**Ligand**	**CAS Number**	**AlinOBP11**	
		**IC_50_ (μM)**	**K_i_ (μM)**
**GENERAL ODORANTS**			
2-Hexanol	626-93-7	u.d.	u.d.
Pentanol	71-41-0	u.d.	u.d.
Valeraldehyde	110-62-3	u.d.	u.d.
Hexanal	66-25-1	u.d.	u.d.
Heptanal	111-71-7	u.d.	u.d.
Octanal	124-13-0	u.d.	u.d.
Nonanal	124-19-6	u.d.	u.d.
2-Hexanone	591-78-6	u.d.	u.d.
2-Heptanone	110-43-0	u.d.	u.d.
2-Octanone	111-13-7	u.d.	u.d.
3-Hexanone	589-38-8	u.d.	u.d.
6-Methyl-5-hepten-2-one	110-93-0	u.d.	u.d.
Amyl acetate	628-637-7	u.d.	u.d.
Nonyl acetate	1143-13-5	u.d.	u.d.
Undecane	1120-21-4	u.d.	u.d.
Indole	120-72-9	u.d.	u.d.
Benzaldehyde	100-52-7	u.d.	u.d.
3,4-Dimethyl-benzaldehyde	5973-71-7	u.d.	u.d.
Acetophenone	98-86-2	u.d.	u.d.
Methyl salicylate	119-36-8	u.d.	u.d.
**GREEN LEAF VOLATILES**			
1-Hexanol	111-27-3	u.d.	u.d.
*cis*-3-Hexen-1-ol	928-96-1	u.d.	u.d.
*trans*-2-Hexenal	6278-26-3	u.d.	u.d.
*cis*-3-hexenyl acetate	3681-71-8	u.d.	u.d.
**TERPENOIDS**			
E-β-Ocimene	3016-19-1	u.d.	u.d.
Limonene	5989-27-5	u.d.	u.d.
α-Phellandrene	99-83-2	26.16 ± 0.52	20.07 ± 0.41
β-Pinene	18172-67-3	u.d.	u.d.
(+)-α-Pinene	7785-70-8	u.d.	u.d.
β-Ionone	79-77-6	u.d.	u.d.
Myrcene	123-35-3	u.d.	u.d.
Nerolidol	7212-44-4	27.23 ± 0.71	20.76 ± 0.55
β-Caryophyllene	87-44-5	u.d.	u.d.
α-Humulene	6753-98-6	u.d.	u.d.
*trans*-β-Farnesene	18794-84-8	u.d.	u.d.
*trans,trans*-Farnesol	106-28-5	25.21 ± 2.36	19.26 ± 1.78
**PUTATIVE SEX PHEROMONES**			
Hexyl butyrate	2639-63-6	u.d.	u.d.
Hexyl hexanoate	6378-65-0	u.d.	u.d.
Butyl butyrate	109-21-7	u.d.	u.d.
Ethyl butyrate	105-54-4	u.d.	u.d.
*trans*-2-hexenyl butyrate	53398-83-7	u.d.	u.d.
**HOST PLANT SECONDARY METABOLITES**			
(−)-Catechin	18829-704	19.26 ± 0.93	15.26 ± 0.70
Rutin hydrate	207671-50-9	9.89 ± 1.61	7.78 ± 1.23
Quercetin	117-39-5	3.36 ± 0.30	2.63 ± 0.23
Gossypol	303-45-7	4.45 ± 0.34	3.43 ± 0.32

*U.d. means that the IC_50_ value exceeds 30 μM and thus that the binding affinities (Ki) of the candidate competitive ligand were not calculated in this study*.

## Discussion

Insect OBPs may serve as important molecular target for designing and screening new effectively behavioral blocking agents used in the application of eco-friendly pest management strategies as they are considered to be strongly expressed in antennal sensillum lymph and are involved in olfactory cues discrimination, binding and transduction (Qiao et al., 2009; He et al., 2011; Sun Y. F. et al., 2012; Pelosi et al., 2013, 2014; Sun L. et al., 2013; Sun et al., 2014a). However, a plenty of studies suggested that OBPs' expression patterns are not restricted in olfactory organs and thus their physiological functions would be more complex and diversified (Park et al., 2000; Foret and Maleszka, 2006; Li et al., 2008; Sun Y. F. et al., 2012; Yuan et al., 2015). To confirm whether OBPs in the Hemiptera mirid bug species could fulfill putative gustatory function, in the present study we especially focus on a putative non-olfactory organ biased OBP gene, the *OBP11* in *A. lineolatus*.

Previously, Gu et al. identified 14 putative OBP genes from the antennal cDNA library of *A. lineolatus* and suggested *AlinOBP11* was strongly expressed at adult legs of both sexes (Gu et al., 2011a). Subsequently, a large number of potential OBP genes were identified in the tarnished plant bug, *L. lineolaris* and the green plant bug, *Apolygus lucorum* via transcriptome strategy, and more OBP transcripts were found to be expressed at gustatory organs such as legs and mouthparts (Hull et al., 2014; Yuan et al., 2015). Therefore, we firstly re-confirmed the tissue expression profiles of *AlinOBP11* after taking mouthparts into account, the most important gustatory organs of Hemiptera species. The results of our western blot analysis revealed that clear single bands could be seen at mouthparts, legs as well as antennae of both male and female adult bugs (Figure 2). Interestingly, we found that relative mRNA level of *AlinOBP11* was extraordinarily higher at adult mouthparts of both sexes than that of previous reported legs and other tissues (Figure 3). In addition, higher expression level was also observed in adult bugs than different instars of nymph (Figure 3). If we considered the tissue distribution patterns of all the 14 identified OBP genes, according to the inference that mRNA expression is indicative of physiological function of its encoded protein, a putative functional subdivision of different OBP genes in the same species of *A. lineolatus* would occur and the mouthparts-biased *AlinOBP11* could be separated from other OBPs such as *AlinOBP1, 10, 13* which have been demonstrated strongly expressed at antennae sensillum and fulfilled vital roles in bug olfactory cue perception (Gu et al., 2011b; Sun L. et al., 2013; Sun et al., 2014a). Indeed, unlike the antennae which are equipped with various olfactory sensilla (Chinta et al., 1997; Sun et al., 2014b), mouthparts of Hemiptera bug species consist of piercing-sucking stylets and labium, the former is used to eject saliva for food ingestion and is considered to be directly related to oviposition behavior (Romani et al., 2005), while the latter has 11–12 uniporous gustatory sensilla which are responsible for assessing the suitability of food substrates (Ave et al., 1978; Hatfield and Frazier, 1980). Our current results of tissue expression pattern merely confirmed that *AlinOBP11* was preferentially expressed at *A. lineolatus* adult mouthparts. Although it is not known whether *AlinOBP11* was expressed in stylets or the gustatory sensillum of labium, we can conceivably speculate that this provocatively specific expression profile would benefit the alfalfa plant bugs, to a great extent, in many importantly behavioral performances such as egg laying, host plants selection, and even of toxic substances avoidance.

Our fluorescence competition assay provides further insight into understanding of physiological roles of AlinOBP11. The results clearly showed that recombinant AlinOBP11 protein displayed preferential binding abilities to tested non-volatile host plant secondary metabolites than all the volatile compounds (Figures 4B,C; Table 1). These results correspond well to its specific tissue distribution within mouthparts which could give a functional implication that AlinOBP11 could function as carrier in gustatory system for non-volatile compounds detection when plant bugs begin to search suitable food substrates by using the mouthparts to rub or tap on plant surfaces or insert plant tissues. Additionally, plant secondary compounds play key roles in the long-term evolution of plant-herbivore interactions (Elsayed, 2011; Mithöfer and Boland, 2012), and the content level variation of quercetin, gossypol, and rutin hydrate, three AlinOBP11 best ligands, over the course of host plant maturation have been demonstrated to be involved in herbivore defense. In particular, gossypol and rutin hydrate were proposed to increase the resistance of cotton plants in response to mirid bug feeding, while the content of quercetin in cotton tended to perform a negatively correlation between their interactions (Lin et al., 2011). Thus, *A. lineolatus* might employ the mouthparts-biased expressed AlinOBP11 to perceive and discriminate these functional different non-volatile secondary metabolites; however, this speculation still needs to be supported by more evidences.

Gene duplication was pointed to be the main mechanism underlying the fast expansion and functional evolution of chemosensory genes (Zhou et al., 2010; Zhang and Löfstedt, 2013); nevertheless, physiological functions of putative orthologs also attract great interest. In aphid species, the distribution of orthologous *OBP* genes may reflect their life styles and host relationships. As an example, homologous OBP3 proteins of different aphid species were proved to be associated with recognition of alarm pheromone (*E*)-ß-farnesene (Qiao et al., 2009; Vandermoten et al., 2011; Sun Y. F. et al., 2012). We re-constructed the phylogenetic trees used reported OBPs of several bug species (Figure 1) and the results clearly suggest AlinOBP11 and AsutOBP11, AlucOBP36, AfasOBP11, and LylinOBP19 fall into the same clade and support they are potential orthologs across bug species which was consistent with Hull's assumption (Hull et al., 2014). Selective pressure assess by calculation of dN/dS or ω = 0.295 (Figure 1) also indicates that genes in this cluster are under purifying selection and would perform conserved functions (Qiao et al., 2009; Zhou et al., 2010; Vandermoten et al., 2011). Meanwhile, we found the *AlinOBP11* was predominately expressed at mouthparts similar to tissue expression profiles of previous reported *LylinOBP19* (Hull et al., 2014) and our further studies of *AfasOBP11* and *AsutOBP11* (data not shown here). However, Hua et al. (2012) suggested that *AlucOBP36* (named as *AlucOBP3* in their study) was antennae-biased expressed. This could be explained by different genetic relationships and evolutionary processes of these bugs. *A. lineolatus, A. suturalis*, and *A. fasciaticollis* belong to the same genus *Adelphocoris*, while *A*. *lucorum* belongs to the other genus *Apolygus*. Notably, the *in vitro* functional studies of antennae expressed AlucOBP36 resembled our results of AlinOBP11, which also showed better binding abilities to non-volatile host plant secondary compounds of rutin hydrate, but not to quercetin and gossypol (Hua et al., 2012), and this could be attributed to the mutations of several amino acids in these two proteins' binding pockets.

In conclusion, this study characterizes a mouthparts enriched *OBP11* protein in *A. lineolatus* which preferentially binds to non-volatile plant secondary compounds; to our current knowledge, AlinOBP11 represents the first physiological function of mouthparts highly expressed OBP in Hemiptera species. As putative orthologous genes probably exhibited conserved physiological function, orthologous OBP11 could be involved in mirid bug feeding behaviors and serve as potential molecular targets for the development of eco-friendly pest management strategies against mirid bugs' outbreaks.

## Author contributions

LS and YZ conceived and designed the experimental plan. LS, XM, and YX preformed the experiments. LS, YW, DZ, YZ, XY, QX, and YG analyzed the data. LS and DZ drafted the manuscript.

### Conflict of interest statement

The authors declare that the research was conducted in the absence of any commercial or financial relationships that could be construed as a potential conflict of interest.

## Supplementary material

Figure S1 **SDS-PAGE analyses of AlinOBP11 expression and purification**. Protein markers are shown in the left side; −, crude bacterial extract before induction with IPTG; + crude bacterial extracts after induction with IPTG; Sup, supernatant of disrupted PET/AlinOBP11; Pel, inclusion body of disrupted PET/AlinOBP11; P/His-tag, purified AlinOBP11 protein with His-tag; P, finally purified AlinOBP11 protein obtained after two rounds of purification.

Figure S2 **The relative transcript levels of *AlinOBP11* at different developmental stages and adult tissues of both sexes evaluated by qRT-PCR with *AlinElongation factor* (GenBank No.AEY99651) as internal control**. The results clearly showed *AlinOBP11* was strongly expressed at adult mouthparts.

Table S1 **The primers used in this article**.

Table S2 **The protein names, GenBank accession numbers, and references of OBPs used in the phylogenetic analysis**.
