# Supplementary figures and images for: The Mouthparts Enriched Odorant Binding Protein 11 of the Alfalfa Plant Bug Adelphocoris lineolatus Displays a Preferential Binding Behavior to Host Plant Secondary Metabolites
Source: Front Physiol. 2016 Jun 1;7:201. doi: 10.3389/fphys.2016.00201 (PMC4887496; doi:10.3389/fphys.2016.00201)

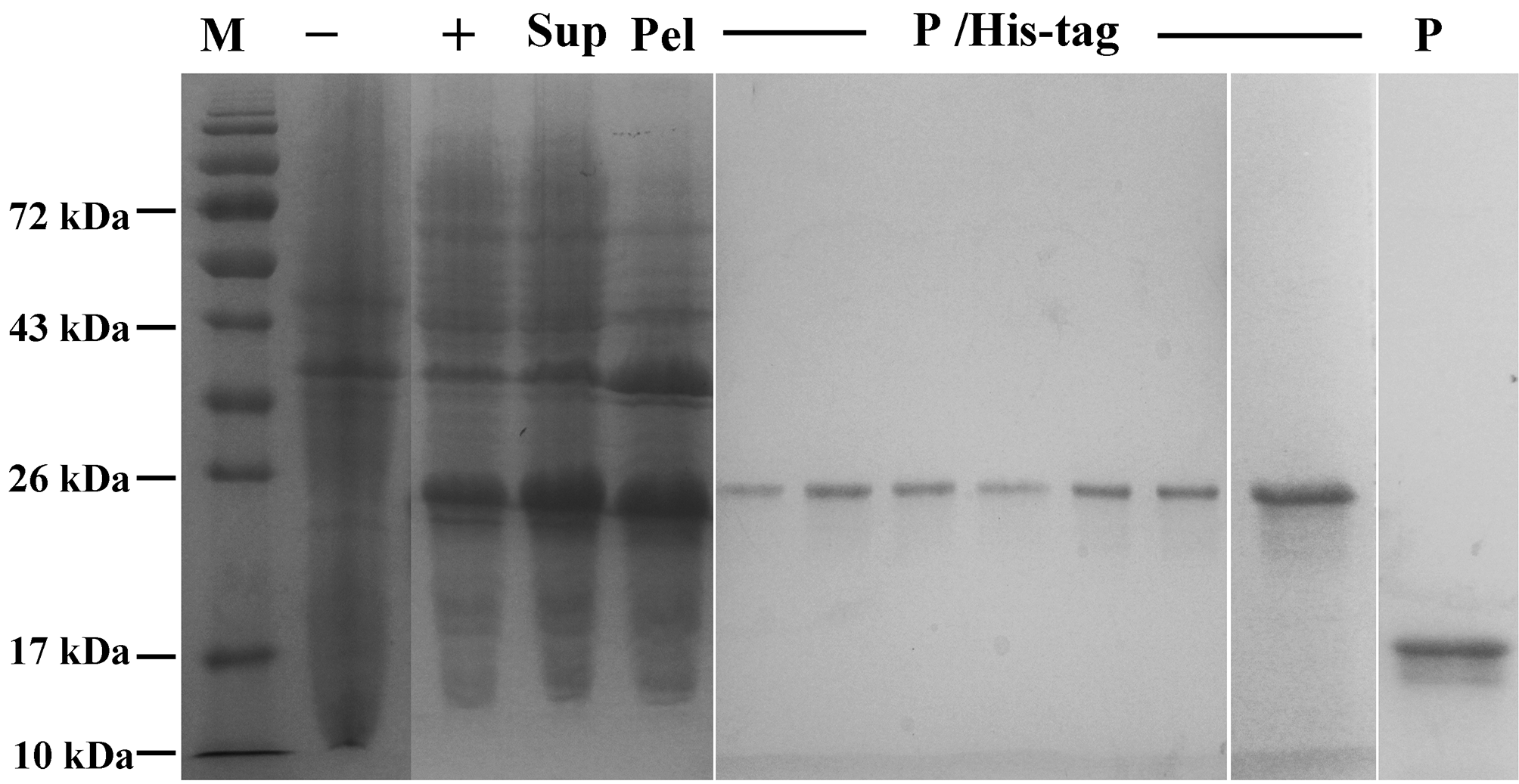

Supplement: Figure S1 — SDS-PAGE analyses of AlinOBP11 expression and purification. Protein markers are shown in the left side; −, crude bacterial extract before induction with IPTG; + crude bacterial extracts after induction with IPTG; Sup, supernatant of disrupted PET/AlinOBP11; Pel, inclusion body of disrupted PET/AlinOBP11; P/His-tag, purified AlinOBP11 protein with His-tag; P, finally purified AlinOBP11 protein obtained after two rounds of purification. [file Image1.TIF]

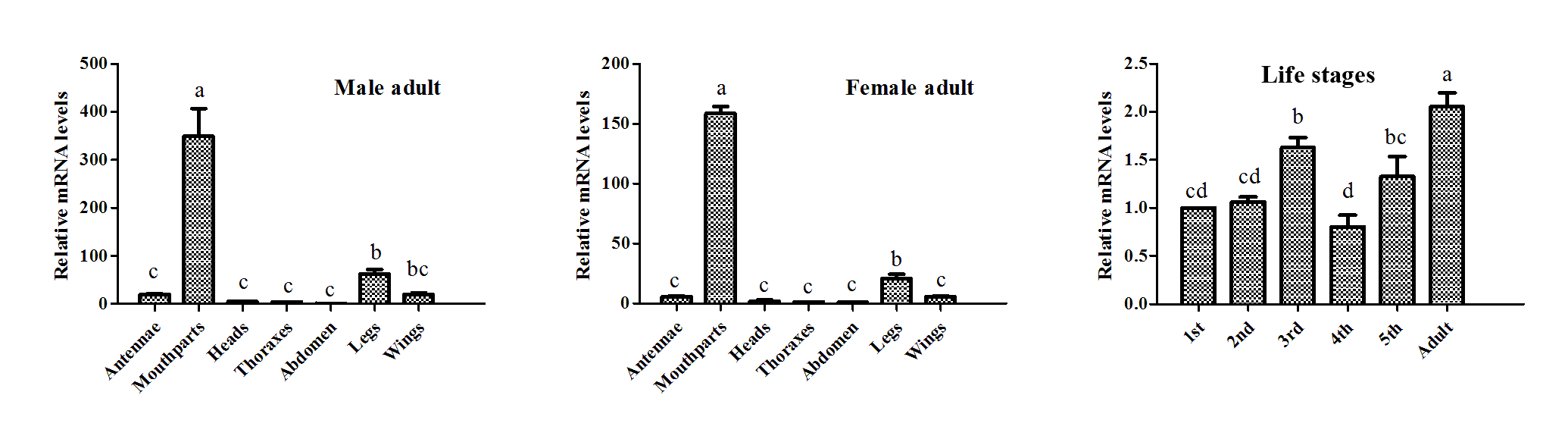

Supplement: Figure S2 — The relative transcript levels of AlinOBP11 at different developmental stages and adult tissues of both sexes evaluated by qRT-PCR with AlinElongation factor (GenBank No.AEY99651) as internal control. The results clearly showed AlinOBP11 was strongly expressed at adult mouthparts. [file Image2.TIF]
