# Supplementary material for: The Mouthparts Enriched Odorant Binding Protein 11 of the Alfalfa Plant Bug Adelphocoris lineolatus Displays a Preferential Binding Behavior to Host Plant Secondary Metabolites
Source: Front Physiol. 2016 Jun 1;7:201. doi: 10.3389/fphys.2016.00201 (PMC4887496; doi:10.3389/fphys.2016.00201)
Supplement: Table S1 — The primers used in this article. [file Table1.DOCX]

Table S1. Primers used in qRT-PCR and recombinant protein expression

| Primer name | Sequence (5'-3') |
| --- | --- |
| **qRT-PCR** |  |
| *AlinOBP11*-Sense | TGGTGGTTGACGGGAAGGT |
| *AlinOBP11*-Anti-sense | CCATTCGCACTCGTTCTCAGT |
| *AlinOBP11*-Probe | FAM-CCGACGCGTGGAAAGCATCTAACAA-TAMRA |
| *Alinβ-actin*-Sense | ACCACCATGTACCCCGGAAT |
| *Alinβ-actin*-Anti-sense | CCGATCCATACGGAGTATTTGC |
| *Alinβ-actin*-Probe | FAM-ATCACCGCTCTTGCCCCATCCA-TAMRA |
| *AlinElongation factor*-Sense | ATTCCCCGGAGACCCACTT |
| *AlinElongation factor*- Anti-sense | CAAGATATTGCGTAAGATCAGGGAG |
| *AlinElongation factor*- Probe | FAM-CGGCACCAAGCCCTACACCATCG-TAMRA |
| **Protein expression** |  |
| AlinOBP11-Sense | TGccatggCAATCAGCAAAGAATACCACGATAAAG |
| AlinOBP11-Anti-sense | TGctcgagTTATCGCCTTTTTGGGTGTTCC |

Primers of *AlinOBP11* and *Alinβ-actin* used in qRT-PCR cited Gu et al., (2011).
