# Supplementary material for: The Mouthparts Enriched Odorant Binding Protein 11 of the Alfalfa Plant Bug Adelphocoris lineolatus Displays a Preferential Binding Behavior to Host Plant Secondary Metabolites
Source: Front Physiol. 2016 Jun 1;7:201. doi: 10.3389/fphys.2016.00201 (PMC4887496; doi:10.3389/fphys.2016.00201)
Supplement: Table S2 — The protein names, GenBank accession numbers, and references of OBPs used in the phylogenetic analysis. [file Table2.DOCX]

Table S2. GenBank accession numbers and references of the OBPs used in the phylogenetic tree.

| **Odorant binding protein name** | **Accession no.** | **Reference** | | |
| --- | --- | --- | --- | --- |
| ***Adelphocoris lineolatus* (Goeze)** |  | | | Gu et al., 2011 |
| AlinOBP1 | GQ477022 | | |  |
| AlinOBP2 | GQ477023 | | |  |
| AlinOBP3 | GQ477024 | | |  |
| AlinOBP4 | GQ477025 | | |  |
| AlinOBP5 | GQ477026 | | |  |
| AlinOBP6 | GQ477027 | | |  |
| AlinOBP7 | GQ477028 | | |  |
| AlinOBP8 | GQ477029 | | |  |
| AlinOBP9 | GQ477030 | | |  |
| AlinOBP10 | GQ477031 | | |  |
| AlinOBP11 | GQ477032 | | |  |
| AlinOBP12 | GQ477033 | | |  |
| AlinOBP13 | GQ477034 | | |  |
| AlinOBP14 | GQ477035 | | |  |
| ***Adelphocoris fasciaticollis* Reuter** |  | |  | |
| AfasOBP11 | KU230352 | | NCBI | |
| ***Adelphocoris suturalis* Jakovlev** |  | |  | |
| AsutOBP6 | KF921002 | | NCBI | |
| AsutOBP7 | KF921000 | |  |  |
| AsutOBP8 | KF921003 | |  |  |
| AsutOBP10 | KF921001 | |  |  |
| AsutOBP11 | KF921004 | |  |  |
| AsutOBP12 | KF921005 | |  |  |
| ***Apolygus lucorum* (Meyer-Dür)** |  | |  | |
| AlucOBP1 | HQ631397 | | Ji et al., 2013;  Yuan et al., 2015 | |
| AlucOBP2 | HQ631398 | |  |  |
| AlucOBP3 | HQ631399 | |  |  |
| AlucOBP4 | HQ631400 | |  |  |
| AlucOBP5 | HQ631401 | |  |  |
| AlucOBP6 | HQ631402 | |  |  |
| AlucOBP7 | JQ675724 | |  |  |
| AlucOBP8 | JQ675725 | |  |  |
| AlucOBP9 | JQ675726 | |  |  |
| AlucOBP10 | JQ675727 | |  |  |
| AlucOBP11 | JQ675728 | |  |  |
| AlucOBP12 | JQ675729 | |  |  |
| AlucOBP13 | KT281921 | |  |  |
| AlucOBP14 | KT281922 | |  |  |
| AlucOBP15 | KT281923 | |  |  |
| AlucOBP16 | KT281924 | |  |  |
| AlucOBP17 | KT281925 | |  |  |
| AlucOBP18 | KT281926 | |  |  |
| AlucOBP19 | KT281927 | |  |  |
| AlucOBP20 | KT281928 | |  |  |
| AlucOBP21 | KT281929 | |  |  |
| AlucOBP22 | KT281930 | |  |  |
| AlucOBP23 | KT281931 | |  |  |
| AlucOBP24 | KT281932 | |  |  |
| AlucOBP25 | KT281933 | |  |  |
| AlucOBP26 | KT281934 | |  |  |
| AlucOBP27 | KT281935 | |  |  |
| AlucOBP28 | KT281936 | |  |  |
| AlucOBP29 | KT281937 | |  |  |
| AlucOBP30 | KT281938 | |  |  |
| AlucOBP31 | KT281939 | |  |  |
| AlucOBP32 | KT281940 | |  |  |
| AlucOBP33 | KT281941 | |  |  |
| AlucOBP34 | KT281942 | |  |  |
| AlucOBP35 | KT281943 | |  |  |
| AlucOBP36 | KT281944 | |  |  |
| AlucOBP37 | KT281945 | |  |  |
| AlucOBP38 | KT281946 | |  |  |
| ***Lygus lineolaris*** |  | |  | |
| LylinOBP1 | KF240735 | | Hull et al., 2014 | |
| LylinOBP2 | KF240736 | |  |  |
| LylinOBP3 | KF240737 | |  |  |
| LylinOBP4 | KF240738 | |  |  |
| LylinOBP5 | KF240739 | |  |  |
| LylinOBP6 | KF240740 | |  |  |
| LylinOBP7 | KF240741 | |  |  |
| LylinOBP8 | KF240742 | |  |  |
| LylinOBP9 | KF240743 | |  |  |
| LylinOBP10 | KF240744 | |  |  |
| LylinOBP11 | KF240745 | |  |  |
| LylinOBP12 | KF240746 | |  |  |
| LylinOBP13 | KF240747 | |  |  |
| LylinOBP14 | KF240748 | |  |  |
| LylinOBP15 | KF240749 | |  |  |
| LylinOBP16 | KF240750 | |  |  |
| LylinOBP17 | KF240752 | |  |  |
| LylinOBP18 | KF240753 | |  |  |
| LylinOBP19 | KF240756 | |  |  |
| LylinOBP20 | KF240757 | |  |  |
| LylinOBP21 | KF240758 | |  |  |
| LylinOBP22 | KF240759 | |  |  |
| LylinOBP23 | KF240760 | |  |  |
| LylinOBP24 | KF240762 | |  |  |
| LylinOBP25 | KF240763 | |  |  |
| LylinOBP26 | KF240764 | |  |  |
| LylinOBP27 | KF240765 | |  |  |
| LylinOBP28 | KF240766 | |  |  |
| LylinOBP29 | KF240767 | |  |  |
| LylinOBP30 | KF240768 | |  |  |
| LylinOBP31 | KF240769 | |  |  |
| LylinOBP32 | KF240770 | |  |  |
| LylinOBP33 | KF240761 | |  |  |
